# Cure of Scabies by Inunction with Lard

**Published:** 1850-09

**Authors:** 


### ARTICLE VII.

*Cure of Scabies by Inunction with Lard.*—Dr. Bennett has confirmed the assertion made by some Italian physicians, that Sulphur ointment cures itch only in virtue of its oleaginous ingredent, which asyhyxiates the acarus by stopping up its spiracula. Four cases are given in which the disease disappeared by the simple use of hog's lard.---*Ed. Monthly Jour.*

